# Supplementary material for: Prevalence of Mental Health Disorders among Elderly Diabetics and Associated Risk Factors in Indonesia
Source: Int J Environ Res Public Health. 2021 Sep 30;18(19):10301. doi: 10.3390/ijerph181910301 (PMC8508332; doi:10.3390/ijerph181910301)
Supplement: Supplementary file 1 [file ijerph-18-10301-s001.zip › ijerph-1352822-supplementary.pdf]

Kusioner Ganguan Mental Emosional (RISKESDAS 2018)

Self Reporting Questionnaire 20 (SRQ20) for Mental Health Disorder Screening on RISKESDAS 2018

https://labmandat.litbang.kemkes.go.id/ccount/click.php?id=21

| | | | |
|---|---|---|---|
| | [illegible] | | |
| **KESEHATAN MENTAL EMOSIONAL [KHUSUS UNTUK ART UMUR ≥ 15 TAHUN DAN "TIDAK DIWAKILI"]** | | | |
| Kami akan mengajukan 20 pertanyaan. Kalau kurang mengerti kami akan membacakan sekali lagi, namun kami tidak akan menjelaskan/ mendiskusikan. Jika ada pertanyaan akan kita bicarakan setelah selesai menjawab ke 20 pertanyaan. | | | |
| C12 | **Dalam 1 bulan terakhir**, apakah [NAMA] sering menderita sakit kepala? | 1. Ya 2. Tidak | ☐ |
| C13 | **Dalam 1 bulan terakhir**, apakah [NAMA] tidak nafsu makan? | 1. Ya 2. Tidak | ☐ |
| C14 | **Dalam 1 bulan terakhir**, apakah [NAMA] sulit tidur? | 1. Ya 2. Tidak | ☐ |
| C15 | **Dalam 1 bulan terakhir**, apakah [NAMA] mudah takut? | 1. Ya 2. Tidak | ☐ |
| C16 | **Dalam 1 bulan terakhir**, apakah [NAMA] merasa tegang, cemas atau kuatir? | 1. Ya 2. Tidak | ☐ |



| | | | |
|---|---|---|---|
| C17 | **Dalam 1 bulan terakhir,** apakah tangan [NAMA] gemetar? | 1. Ya 2. Tidak | ☐ |
| C18 | **Dalam 1 bulan terakhir**, apakah pencernaan [NAMA] terganggu/ buruk? | 1. Ya 2. Tidak | ☐ |
| C19 | **Dalam 1 bulan terakhir**, apakah [NAMA] sulit untuk berpikir jernih? | 1. Ya 2. Tidak | ☐ |
| C20 | **Dalam 1 bulan terakhir**, apakah [NAMA] merasa tidak bahagia? | 1. Ya 2. Tidak | ☐ |
| C21 | **Dalam 1 bulan terakhir**, apakah [NAMA] menangis lebih sering? | 1. Ya 2. Tidak | ☐ |
| C22 | **Dalam 1 bulan terakhir**, apakah [NAMA] merasa sulit untuk menikmati kegiatan sehari-hari? | 1. Ya 2. Tidak | ☐ |
| C23 | **Dalam 1 bulan terakhir**, apakah [NAMA] sulit untuk mengambil keputusan? | 1. Ya 2. Tidak | ☐ |
| C24 | **Dalam 1 bulan terakhir**, apakah pekerjaan [NAMA] sehari-hari terganggu? | 1. Ya 2. Tidak | ☐ |
| C25 | **Dalam 1 bulan terakhir**, apakah [NAMA] tidak mampu melakukan hal-hal yang bermanfaat dalam hidup? | 1. Ya 2. Tidak | ☐ |
| C26 | **Dalam 1 bulan terakhir**, apakah [NAMA] kehilangan minat pada berbagai hal? | 1. Ya 2. Tidak | ☐ |
| C27 | **Dalam 1 bulan terakhir**, apakah [NAMA] merasa tidak berharga? | 1. Ya 2. Tidak | ☐ |
| C28 | **Dalam 1 bulan terakhir**, apakah [NAMA] mempunyai pikiran untuk mengakhiri hidup? | 1. Ya 2. Tidak | ☐ |
| C29 | **Dalam 1 bulan terakhir**, apakah [NAMA] merasa lelah sepanjang waktu? | 1. Ya 2. Tidak | ☐ |
| C30 | **Dalam 1 bulan terakhir**, apakah [NAMA] mengalami rasa tidak enak di perut? | 1. Ya 2. Tidak | ☐ |
| C31 | **Dalam 1 bulan terakhir**, apakah [NAMA] mudah lelah? | 1. Ya 2. Tidak | ☐ |

Translated from WHO SRQ-20

| **SRQ-20** |
| --- |
| Headaches |
| Poor appetite |
| Sleeping badly |
| Easily frightened |
| Nervous, tense, worried |
| Hands shaking |
| Poor digestion |
| Trouble thinking clearly |
| Feeling unhappy |
| Crying more often |
| Difficult enjoying activities |
| Difficult making decisions |
| Daily work suffering |
| Unable to play useful part |
| Lost interest in things |
| Feeling worthless |
| Thought of ending your life been in your mind |
| Feeling always tired |
| Uncomfortable feelings in stomach |
| Easily tired |
